# Cause rare d’insuffisance rénale aigue: géant calcul de vessie chez une jeune femme: A rare cause of acute renal failure: giant urinary bladder stone in a young woman

**DOI:** 10.11604/pamj.2018.29.4.14308

**Published:** 2018-01-03

**Authors:** Bounoual Mohammed, Ahsaini Mustapha, Tazi Karim, Azelmad Hamid, Alila Mohammed, Mellas Soufiane, El Ammari Jalal eddine, Tazi Mohammed Fadl, El Fassi Mohammed Jamal, Farih Moulay Hassan

**Affiliations:** 1 Service d’Urologie, Centre Hospitalier Universitaire Hassan II de Fès, Maroc; 2 Service de Chirurgie Viscérale A, Centre Hospitalier Universitaire Hassan II de Fès, Maroc

**Keywords:** Géant calcul de vessie, insuffisance rénale, idiopathique, cystolithotomie, Giant urinary bladder stone, renal failure, idiopathic, cystolithotomy

## Abstract

Géant calcul de vessie se définit par un poids supérieur à 100 g, c'est une pathologie rare chez la femme, environ 2%. La localisation vésicale ne dépasse pas 5% de l'ensemble des voies urinaires. Chez la femme l'apparition de calcul de vessie est souvent secondaire à un facteur prédisposant notamment un corps étranger intra vésical, vessie neurogene, les infections urinaires à répétition, ATCDS de chirurgie de l'incontinence urinaire d'effort. Mais on retrouve des calculs de vessie sans causes évidentes dits calculs primaires idiopathiques. On rapporte un cas d'une jeune patiente de 31 ans avec un géant calcul de vessie qui a retenti sur le haut appareil urinaire entrainant une insuffisance rénale aigue. La patiente a bénéficié initialement d'un drainage de haut appareil urinaire par des néphrostomies bilatérales puis elle a bénéficié d'une cystolithotomie à ciel ouvert d'un gros calcul de vessie. Un bilan étiologique a été demandé pourtant n'a pas identifié de cause évidente.

## Abstract (en)

Giant urinary bladder stone is defined as a stone with a weight higher than 100 g. It is rare in women, accounting for approximately 2%. Its occurrence in the urinary bladder does not exceed 5% of the whole of the urinary tract. In women, the occurrence of urinary bladder stone is often secondary to a predisposing factor including intravesical foreign body, neurogenic bladder, repeated urinary infections, a history of surgery treating stress urinary incontinence. Therefore, there are some patients with a bladder stones without any obvious cause, such as primary idiopathic stone. We here report the case of a 31-year old patient with giant urinary bladder stone affecting the superior part of the urinary tract causing acute renal failure. The patient initially underwent bilateral nephrostomies to drain the superior part of the urinary tract; then giant urinary bladder stone was removed by open cystolithotomy. Etiological assessment didn't show any obvious cause.

## Introduction

Le calcul de vessie est une pathologie rare chez la femme, il est souvent secondaire d'où la nécessité d'un bilan étiologique bien conduit [1-3]. Un géant calcul peut causer des dégâts sur le haut appareil avec hydronéphrose et insuffisance rénale aigue [4]. Le traitement dépend de la taille de calcul, ainsi une cystolithotomie par laparotomie sous ombilicale est indiquée pour les gros calculs géants [5, 6], nous rapportant un cas d'une jeune patiente présentant un géant calcul avec insuffisance rénale aigue chez qui des examens complémentaires n'avaient pas objectivé d'étiologie. Une extraction chirurgicale de calcul a été réalisée après drainage de haut appareil.

## Patient et observation

**Figure 1:**
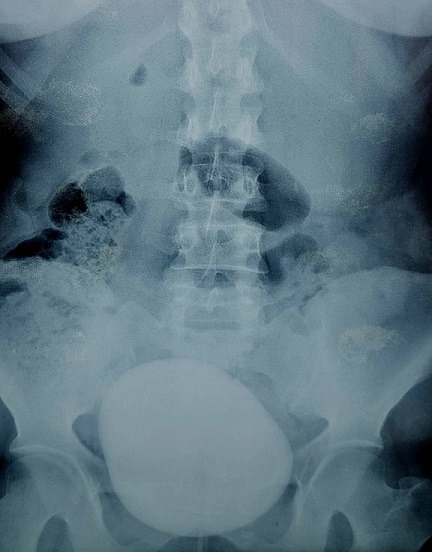
Arbre urinaire sans préparation montrant une opacité de tonalité calcique se projetant sur l’aire vésicale

**Figure 2:**
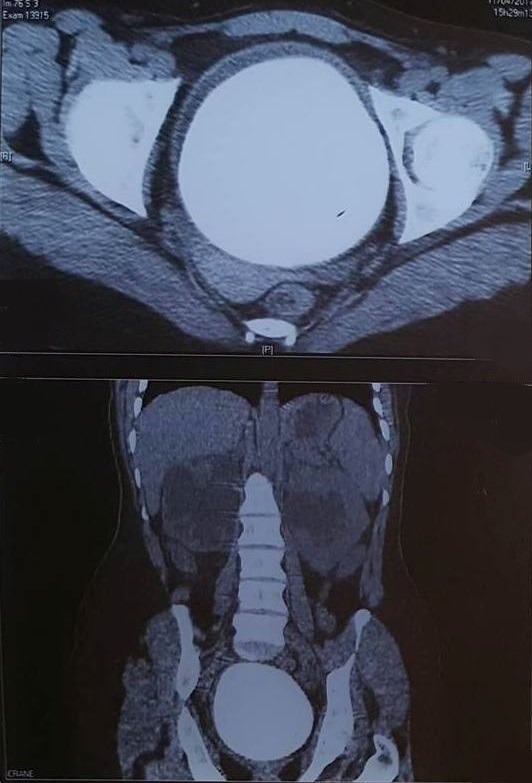
TDM abdomino-pelvienne montrant un énorme calcul de vessie avec dilatation urétéropyelocalicielle en amont

**Figure 3:**
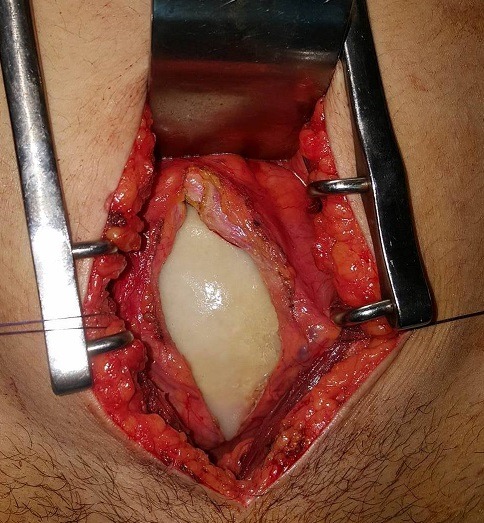
Vue opératoire montrant le calcul de vessie à travers la cystotomie

**Figure 4:**
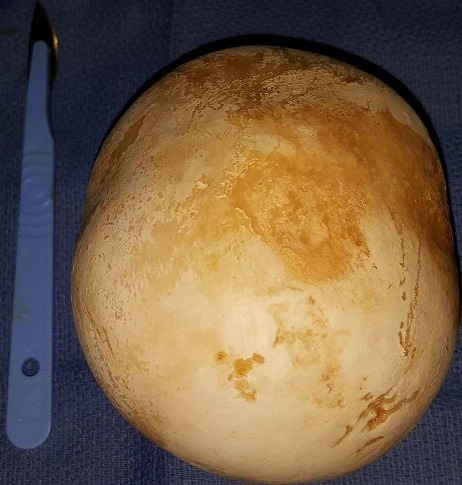
Géant calcul de vessie faisant 10cm de grand axe

Il s'agit d'une patiente de 31 ans, sans antécédents pathologiques notables, qui s'est présentée aux urgences pour des douleurs pelviennes et lombaire en bilatérales avec une dysurie et brulures mictionnels évoluant dans un contexte de fièvre non chiffrée, l'examen clinique trouve une patiente altérée fébrile à 39 degré, l'examen abdominale avait révélé une masse dure en sus pubien avec une sensibilité lombaire bilatérale. Un bilan biologique avait montré une insuffisance rénale aigue avec une créatinine à 52 mg/l, urée: 1,26 g/l, un syndrome inflammatoire avec CRP à 256mg/l, leucocyte à 12000 éléments/l. L'examen des urines montre une infection urinaire à providencia rettgeri sensible au ceftriaxone. Un arbre urinaire sans préparation (AUSP) (Figure 1) avait objectivé une grosse image de tonalité calcique se projetant sur la vessie évoquant un géant calcul vésical. Une échographie ainsi une TDM abdomino-pelvienne sans injection de produit de contraste (Figure 2) avait objectivé une urétéro-hydronéphrose bilatérale en amont d'un gros calcul de vessie faisant 10,3cm de grand axe. La patiente a bénéficié d'une antibiothérapie parentérale à base de ceftriaxone à la dose de 2g/24h, puis deux néphrostomies sont réalisées en urgences. Après normalisation de la fonction rénale et refroidissement par une antibiothérapie, une cystolithotomie par une incision médiane sus pubienne est réalisée permettant l'extraction d'un gros calcul de 10cm et 700g de poids (Figure 3, Figure 4), sa nature spectrophotometrique est phosphoamoniaque et oxalate de calcium, le Redon a été enlevé le lendemain et la patiente est sortie d'hôpital 48h en post opératoire. La sonde vésicale est enlevée 10 jours après le geste. Le bilan biologique de contrôle s'est normalisé, ainsi une échographie vésicale n'avait pas objectivé de résidu post mictionnel. Le débitmètre avait montré une capacité vésicale à 500 cc, Q max à 15 et une courbe mictionnelle en cloche tout à fait normale puis la cystoscopie n'a pas montré de sténose sous vésicale, le col vésical s'ouvre normalement, la vessie distendue à muqueuse normales. On a réalisé un examen urodynamique qui n'a pas révélé d'anomalie particulière.

## Discussion

Les calculs de vessie se développent rarement chez la femme [1-3], la localisation vésicale ne dépasse pas 5% de l'ensemble de tractus urinaire [7]. Les troubles urinaires de bas appareil notamment la dysurie, la pollakiurie, les brulures mictionnelles, les douleurs pelviennes et parfois l'hématurie sont non spécifiques mais aident au diagnostic positif. L'examen physique n'est utile que pour les gros calculs géants palpables en sus pubien ou aux touchers pelviens [3, 6-8]. L'imagerie urinaire: AUSP, échographie et surtout le scanner abdomino-pelvien constitue l'examen clé au diagnostic et aussi à l'évaluation de retentissement sur le haut appareil et l'identification de l'étiologie. Les calculs sans causes évidentes dits calcul primaire idiopathique [7], rare c'est le cas de notre patiente, mais revenant à la littérature, il est souvent secondaire notamment à un corps étranger intra vésical (fragment de ballonnet de la sonde vésical [3], migration vésicale d'un dispositif intra utérin [9] ou iatrogène après une chirurgie pelvienne ou chirurgie de l'incontinence urinaire (obstruction sous vésicale, fils non résorbable [2]). Les infections urinaires chroniques sont évoquées dans les calculs infectieux phospho-amoniaco-magnésium [8]. Les vessies neurogenes peuvent aussi être une cause de calcul au dépend d'une stase urinaire vésicale. Les gros calculs qui mêlent toute la lumière vésicale exercent une compression mécanique sur les méats urétéraux ainsi ils entrainent une obstruction sur les voies urinaires supérieures avec développement d'une insuffisance rénale obstructive [4], c'est un incident rare et c'est le cas de nôtre patiente. Le traitement commence tout d'abord par la correction des complications infectieuses et obstructives de ce calcul, notamment une antibiothérapie adaptée avec un drainage des voies urinaires hautes par une néphrostomie bilatérale, vue l'impossibilité de réaliser une montée de sonde urétérale gênée surtout par le calcul qui occupe toute la lumière vésicale [4, 8]. La technique d'extraction d'un calcul vésical dépend de sa taille, ses compositions, les comorbidités de patients et ses ATCDS chirurgicaux, la présence ou non de malformation anatomique de bas appareil urinaire ainsi un calcul de moins de 2cm recommande une extraction par voie endoscopique après fragmentation, pour les calculs de 2 à 4cm une cystolithotomie par voie percutanée est préconisée alors si le calcul est de plus de 4cm l'extraction chirurgicale par voie ouverte reste la technique la plus recommandée. Une lithotripsie extra corporelle trouve son indication dans les calculs de moins de 2cm chez les patients à haut risque chirurgical avec un taux de succès de 72% à 100%. L'élimination du facteur causal est obligatoire pour le succès thérapeutique [5, 6].

## Conclusion

La lithiase vésicale est une pathologie rare dans la pratique de l'urologue, sa suspicion doit se faire devant des troubles urinaires chroniques de bas appareil chez la femme. Le calcul de vessie est souvent lié à un facteur favorisant mais il y a aussi les calculs primaires idiopathiques. Un géant calcul impose un bilan de retentissement surtout sur le haut appareil, le traitement est chirurgical consiste à extraire le calcul et à corriger les facteurs causals. L'association d'un géant calcul primaire avec une insuffisance rénale aigue est très rare, ce qui fait la particularité de notre observation.

## Conflits d’intérêts

Les auteurs ne déclarent aucuns conflit d'intérêts.
